# Transitions Into Underage and Problem Drinking: Summary of Developmental Processes and Mechanisms: Ages 10–15

**Published:** 2009

**Authors:** Michael Windle, Linda P. Spear, Andrew J. Fuligni, Adrian Angold, Jane D. Brown, Daniel Pine, Greg T. Smith, Jay Giedd, Ronald E. Dahl

**Keywords:** Preadolescent, adolescent, puberty, underage drinking, problematic drinking, risk factors, protective factors, alcohol and other drug (AOD) use initiation, AOD use behavior, growth and development, biological development, psychological development

## Abstract

Adolescents ages 10–15 experience dramatic changes in their biological, cognitive, emotional, and social development as well as in their physical and social environments. These include the physiological and psychological changes associated with puberty; further development of the brain; changes in family, peer, and romantic relationships; and exposure to new societal and cultural influences. During this period, many adolescents also begin to use alcohol. Alcohol use during adolescence has adverse effects on the body and increases the risk of alcohol dependence later in life. To better understand why some children drink whereas others do not, researchers are examining nonspecific and alcohol-specific factors that put adolescents at risk for, or which protect them from, early alcohol use and its associated problems. Nonspecific risk factors include certain temperamental and personality traits, family factors, and nonnormative development. Examples of nonspecific protective factors include certain temperamental characteristics, religiosity, and parenting factors (e.g., parental nurturance and monitoring). Among the most influential alcohol-specific risk and protective factors are a family history of alcoholism and the influences of siblings and peers, all of which shape an adolescent’s expectancies about the effects of alcohol, which in turn help determine alcohol use behaviors.

As described in the preceding article by Zucker and colleagues, even children under the age of 10 can recognize alcohol, begin to form some opinions regarding its use and the consequences of that use, and may even have their first experiences with alcohol. Moreover, various risk and protective factors already are active during this early developmental period that influence drinking behavior during adolescence and adulthood. All of these processes continue during the developmental period from ages 10 to 15, which is the time when many adolescents begin experimenting with alcohol. This article takes a closer look at the relationship between the developmental period from ages 10 to 15 and the use of alcohol. The chapter begins with an overview of normative human development for the age-group (i.e., what is expected at different times and what is typical and atypical at a particular age). It then discusses alcohol use during early and middle adolescence and the risk and protective factors related to underage drinking and to future use.

## Normative Development for Ages 10–15: An Overview

The period from age 10 to 15 years encompasses early adolescence and the beginning of middle adolescence. It is characterized by dramatic changes in biological, cognitive, emotional, and social processes, as well as in physical and social environments. Puberty is a hallmark of this period. Importantly, during the past century the average age of puberty has decreased, with ramifications for adolescent social functioning and behavior. The period from age 10 to 15 also is notable for transitions such as moving from elementary to middle school and from middle school to high school. Moreover, self-identity issues become important during this period, as do peer and societal expectations, including those involving the use of alcohol. In fact, it is the period during which many young people initiate alcohol use and when drinking and binge drinking escalate. The table summarizes the developmental periods, transitions, contexts, tasks, and issues that characterize the 10–15 age-group.

### Characterizing Change in Early and Middle Adolescence

The timing and tempo of various developmental processes varies both within and across individuals. Some changes of early and middle adolescence are closely related to chronological age, such as grade in school. Other changes are more closely aligned with developmental stage, such as puberty, interest in the opposite sex, and the relative importance of peers.

### Puberty

The changes in the secretory patterns of gonadal steroids that define the onset of puberty generally begin by age 10. They are accompanied by changes in the central nervous system and neurophysiology as well as by increasingly apparent changes in physical appearance (e.g., height, body composition, and the appearance of secondary sexual characteristics). However, significant individual variation as well as variation across racial and ethnic groups exists in these processes. For example, breast and pubic hair development in non-Hispanic White girls typically begins around age 10.5, and a year earlier in non-Hispanic Black girls, with Hispanic girls falling in between. The average age at menarche is later, beginning around age 12.5 in non-Hispanic White girls and a few months earlier in non-Hispanic Black girls.

Although the growth spurt associated with puberty occurs later in boys than girls, generalizations to other aspects of puberty can be misleading. For non-Hispanic Whites, the median onset of pubic hair development was found to occur later in boys than girls (12.0 vs. 10.6 years), whereas the median onset of genital development in boys occurred earlier (10.0 years) than the onset of breast development in girls (10.4 years) (Sun et al. 2002).

### Changes in the Developing Brain

During adolescence, a series of maturational changes occurs in the brain as a result of hormonal changes and the accrual of experience. The increase in gray matter that occurs up until about age 11 in girls and age 12 in boys is followed by a gradual decrease in gray matter volume in the cerebral cortex (Giedd et al. 1997, 1999; Gogtay et al. 2004; Sowell et al. 2004; Toga and Thompson 2003). The decrease is thought to result from developmental processes, such as reductions in synaptic connections among neurons known as dendritic “pruning.” The period between ages 10 and 15 years old involves significant growth in cognitive processes, including the ability to plan, maintain information “online,” solve complex cognitive tasks, and exhibit self-regulation and inhibitory control (Luna and Sweeney 2004). Importantly, regulatory control processes continue to develop well past age 15, into a person’s twenties.

Adolescents report greater fluctuations in their emotional states (Larson et al. 2002), tend to experience highly emotional events more acutely, and exhibit more episodes of rule-violating behavior than preadolescents (Moffitt and Caspi 2001). Patterns of sleep and arousal regulation also change substantially in adolescence, with a shift in circadian rhythms stimulating individuals to stay awake longer and sleep until later in the day (Nelson et al. 2002). These changes are associated with broader changes in behavioral regulation, which may underlie risk for the development of various forms of psychopathology. In fact, the rates of many psychopathologies, including major depression, social anxiety disorder, various behavior disorders, and substance use disorders increase significantly among adolescents ages 10–15 (Angold et al. 1998; Costello et al. 2002).

### Changes in Family, Peer, and Romantic Relationships

The most important social relationships for all children and adolescents are those with their families and peers. Between the ages of 10 and 15, these critical relationships undergo dramatic changes.

#### Family Relationships

The belief that young people typically become estranged from their families during adolescence is inaccurate; however, it has been suggested that reaching reproductive maturity triggers an evolutionarily adaptive mechanism that promotes separation from the natal family (Steinberg 1989). Moreover, emotional distancing between children and their parents does increase, particularly between children and their fathers (Fuligni 1998; Steinberg 1988). Early adolescents spend significantly less time with their parents and other family members, which may explain why the overall frequency of parent–child conflict does not increase during these years. The intensity of arguments, however, when they do occur, appears to be greater (Laursen et al. 1998). During this phase, adolescents seek greater social and emotional autonomy and the opportunity to make decisions for themselves (Smetana 1988). They increasingly spend more time with peers (much of which is largely unsupervised by adults) and are more influenced by them. In addition, as their ability to think abstractly increases, adolescents become more proficient at arguing and more critical of their parents, idealizing how they “should” be (Collins 1990). All of these changes may alter the nature of the parent–child relationship, but it is important to note that parents remain a significant influence in their children’s lives.

#### Peer Influence

Peer influence peaks around age 11–13. This is the age range when most American adolescents are in middle school (Berndt 1979; Steinberg and Silverberg 1986) and when frequent class changes and more time spent out of class increase their exposure to peers. An adolescent’s peers, friends, and intimate others typically contribute much more to the development of his or her beliefs, behaviors, choice of leisure activities, and personal preferences (e.g., in clothes and music) than they did prior to age 10. This influence also can extend to risky and antisocial behavior (Berndt 1979). Parents, on the other hand, retain their influence on fundamental issues, such as religion, morality, and education.

Early adolescents who have difficult relationships with their families (e.g., their parents are too controlling or not involved with them) are more likely to orient toward peers (Fuligni and Eccles 1993). Some are willing to sacrifice positive activities and aspects of their lives in order to be accepted by, and be popular with, peers. This may lead them to affiliate with more deviant peer groups and engage in more risky behavior, including alcohol use, during the high school years (Fuligni et al. 2001). Of course, friendship cliques and peer groups are very diverse (Brown 1990), and not all early adolescent peer groups engage in risky behavior.

#### Peer Involvement and Pubertal Maturation

The increase in peer involvement coincides with the peak of pubertal maturation. Whereas this typically occurs between the ages of 10 and 15, there is considerable variation in the tempo and timing of puberty. The attainment of reproductive maturity and the emergence of secondary sex characteristics that distinguish this period can be a source of popularity and status as well as of self-consciousness and concern as adolescents adjust to their maturing bodies and compare themselves to others. For example, studies have shown that early-maturing girls have increased risk for internalizing problems (i.e., anxiety or depression). For some, this may be linked to the increase in body fat that is necessary for menarche to occur (Ge et al. 1996; Peterson 1988). In addition, some early-maturing girls are at higher risk for exposure to peer contexts in which behaviors such as drinking occur because of their affiliation with older boys (Magnusson et al. 1985).

#### Romantic Relationships

Whereas the initiation of dating is influenced by gender, ethnicity, and religiosity (Collins 2003), among other things, adolescents in the United States typically begin to date around the age of 13 or 14. Early-adolescent dating is thought to be more socially than romantically directed and a way to spend time with friends. More serious and enduring romantic relationships typically develop later in adolescence.

On average, today’s adolescents engage in sexual activity at an earlier age and more frequently than in the past. As with dating, there are important variations across gender, ethnicity, and religiosity, and although the majority of youth do not engage in sexual intercourse before the age of 15, those who do may be at elevated risk for drug and alcohol use (Rosenthal et al. 1999).

### Changes in Physical and Family Contexts and Societal and Cultural Influences

For the 10–15 age-group, changes in environmental and social contexts are dramatic, reflecting a broadening of environmental influences. Some contexts assume greater or lesser significance in the adolescent’s life than before, whereas others are relatively new to this age-group. Family and school remain the dominant physical contexts, although, as already mentioned, adolescents gradually spend less time with their parents, a trend that continues throughout high school. Among boys, time with parents and families is replaced by time spent alone and, among girls, by time alone and with friends (Larson and Richards 1991).

For the vast majority of children in the United States, early adolescence spans the transition from elementary school to middle or junior high school. For many, changing schools alone can be stressful. Adding to the stress, the everyday school environment of middle school is dramatically different than that of elementary school (Eccles et al. 1993; Simmons and Blyth 1987). As a result of larger schools and student bodies, multiple classes and teachers, more stringent grading, comparative performance evaluations, less individualized instruction, and lower levels of student–teacher interaction, many early adolescents experience a loss of intrinsic motivation related to school. Their attachment to their school and their enthusiasm for academic pursuits wanes. On the other hand, many young people become more involved in elective activities, such as sports teams or special-interest clubs. These activities open up important new social environments with peers and noncaregiver adults (e.g., athletic coaches, adult leaders of school-related, church, or community activity groups, etc.). These environmental transitions place new demands on the adolescent, requiring adaptation and greater cognitive, emotional, and social control.

Early adolescents typically are given more autonomy by their families and become more immersed in the environment outside the home. They spend more time hanging out in the neighborhood, in movie theaters, parks, and malls (Steinberg 1990). The problematic aspects of some neighborhoods (e.g., high levels of poverty, crime, and antisocial activity) can have a negative impact on development, particularly during the adolescent years when children are exposed to such factors without filtering or monitoring by parents and families (Leventhal and Brooks-Gunn 2000). The impact is greater in the absence of high-quality after-school activities (Pedersen and Seidman 2005). This may be in part because early adolescents who spend more unsupervised time in the neighborhood are more likely to be exposed to older adolescents and young adults engaged in risky behavior, such as alcohol and substance use.

Early adolescents are actively engaged in figuring out who they are and where they fit into the social landscape. Questions arise about the behavioral and stylistic expectations that their peers and the larger society have for them. Ethnicity, gender, and religion become more important as a source of personal identity (Phinney 1990), and, based on these factors, early adolescents may experience differential treatment and expectations and may find themselves pushed toward or away from certain activities (Greene et al. 2006). As adolescents wrestle with issues of identity, they likely will be faced with choices about alcohol use. The decision to drink may be as much a part of trying on different roles as it is a decision to experience the pharmacologic effects of alcohol.

Adolescents spend much of their day tuned to some form of mass media (on average, 6 to 7 hours a day for 11- to 15-year-olds) (Roberts et al. 2004*a*). The media is a significant social and cultural influence, and often it is experienced away from parents with little or no opportunity for adult monitoring, conversation, or interpretation of messages. A majority of early adolescents have a television set in their bedroom, and virtually all have some kind of sound system. They tune in to a range of media catering to their age-group and, in addition, attend to media developed for older teens. Furthermore, this age-group often uses more than one medium simultaneously (e.g., listening to music while surfing the Internet). Adolescents also spend hours each day using technology to communicate with their friends and meeting new ones (e.g., texting, instant messaging) (Roberts et al. 2004*a*,*b*)

Alcohol messages are widespread in the media that adolescents access. Alcohol is the most frequently portrayed food or drink on network television. About two-thirds of prime-time fictional television programs depict alcohol usage at the rate of about eight drinking acts per hour (Mathios et al. 1998). More than one-fourth of the videos on MTV and VH-1 show alcohol use; both of these television channels are favorites of early adolescents (DuRant et al. 1997). Teenage characters frequently are depicted drinking in teen-oriented movies, rarely with negative consequences (Stern 2005).

The mass media may serve as a kind of “super peer” for alcohol use by modeling values and behaviors related to drinking. Studies on the effects of viewing violence on television may help inform our understanding of how adolescents are affected by alcohol portrayals in the media. Those studies have shown that adolescents are more likely to imitate aggressive behavior if it is committed by attractive characters who do not suffer negative consequences. This same pattern may be applicable to positive portrayals of alcohol in the media. Greater exposure to television, music videos, and alcohol advertising in early adolescence has been associated, in some studies, with earlier onset of alcohol use and heavier beer drinking in later adolescence (Stacey et al. 2004).

## Alcohol Use in the 10- to 15-Year-Old Age-Group

### Prevalence of Alcohol Use

Nationally representative survey data on alcohol use is not available for children under the age of 12. On the other hand, a number of nationally representative surveys do collect information on alcohol consumption by adolescents ages 12 and older, including Monitoring the Future (MTF), National Survey on Drug Use and Health (NSDUH), and Youth Risk Behavior Surveillance System (YRBSS). Data from the 2005 MTF study (Johnston et al. 2006) indicates that 41.0 percent of 8th graders surveyed had used alcohol in their lifetime, as had 63.2 percent of 10th graders. Prevalence rates for use in the last 12 months and last 30 days were 33.9 and 17.1 percent, respectively, for 8th graders and 56.7 and 33.2 percent, respectively, for 10th graders. Flavored alcoholic beverages (e.g., alcopops) seem to be popular with this age-group; 35.5 percent of 8th and 57.0 percent of 10th graders reported consuming them at some time in their lives.

According to the MTF data, 19.5 percent of 8th graders and 42.1 percent of 10th graders reported having been drunk in their lifetime, and 10.5 percent of 8th graders and 21 percent of 10th graders reported having consumed five or more drinks on a single occasion in the last 2 weeks. These data suggest an opportunistic pattern of drinking among early adolescents. In other words, when early adolescents drink, they often drink excessively even though most of those who drink do not drink every day. This pattern of drinking is especially dangerous and may adversely affect health and development.

Survey data from MTF also indicate that attitudes and perceptions change across this early adolescent period toward less disapproval of alcohol use. Strong disapproval of alcohol use (one or two drinks) was relatively high among 8th graders (51.2 percent) but lower among 10th graders (38.5 percent). Approximately 57.2 percent of 8th graders and 53.3 percent of 10th graders rated five or more drinks of alcohol once or twice each weekend as a “great risk” for harm. However, when questioned about how much they thought people risked harming themselves (physically or in other ways) by drinking one to two drinks nearly every day, 31.4 percent of 8th graders and 32.6 percent of 10th graders rated this as a “great risk.” In addition, 64.2 percent of 8th graders and 83.7 percent of 10th graders rated alcohol as fairly easy or very easy to get (Johnston et al. 2006).

### Consequences of Early Adolescent Alcohol Use

Although studies on the consequences of adolescent drinking have not focused specifically on this younger age-group, some do include younger adolescents. For example, in one such study where the mean age was 16.9 years, adolescents reported a range of consequences from their drinking, such as passing out, doing things they regretted the next day, and getting into a fight with someone they did not know (Windle and Windle 2005). Adolescent alcohol use may compromise school performance, is associated with tobacco and illicit drug use, and may cause alterations in the structure and function of the developing brain. In addition, early onset of drinking is associated with future problems, including alcohol dependence and other substance abuse (Grant and Dawson 1997; Labouvie et al. 1997).

Alcohol use by early adolescents also is associated with a range of suicidal behaviors, including ideation, attempts, and completions. Data from the National Adolescent Student Health Survey (Windle et al. 1992) indicates that among 10th-grade female abstainers (i.e., who did not drink alcohol in the past 30 days), 33.5 percent had thought about committing suicide and 12.3 percent had attempted it; among light drinkers (i.e., those who drank alcohol on one to five occasions within the last 30 days), 52.0 percent had thought about suicide and 21.4 percent had attempted it; and among moderate/heavy drinkers (i.e., those who drank on six or more occasions in the last 30 days) 63.1 percent had thought about committing suicide and 38.8 percent had attempted it.

Alcohol consumption among early adolescents also is associated with engaging in sexual intercourse and risky sex (e.g., having multiple sexual partners). Among sexually active teens, 26.2 percent of 9th graders and 21.1 percent of 10th graders in the YRBS study reported alcohol or drug use at last sexual intercourse (Eaton et al. 2006).

### Adolescent Sensitivity to Alcohol

Ethical issues prohibit administering alcohol to young people for research purposes, making it difficult to design studies that examine the biological sensitivity of human adolescents to alcohol. However, in a single study conducted several decades ago, 8- to 15-year-old boys were given a dose of 0.5 ml/kg pure ethanol, which induced peak blood alcohol levels (BALs) that were well within the intoxicating range for adults (Eckardt et al. 1998). Yet, the researchers (Behar et al. 1983) found no behavioral signs of intoxication in these youth, noting that few gross behavioral changes occurred in the children after they had received a dose of alcohol that would cause intoxication in adults.

Subsequent animal research has provided support for the observation that adolescents are relatively insensitive to alcohol’s effects on motor impairment. Studies also show that adolescent animals exhibit decreased sensitivity to other effects of alcohol that can serve as cues to limit intake, including social impairment and sedation (Spear and Varlinskaya 2005). In addition, they are less sensitive to certain postintoxication “hangover” effects (Doremus et al. 2003). These insensitivities to alcohol may be particularly pronounced during the early stages of adolescence (Varlinskaya and Spear 2004).

In contrast to their relative insensitivity to many of the aversive effects of alcohol, adolescent animals are more sensitive than adults to some of its pleasurable effects, including the social facilitation seen at low doses (Varlinskaya and Spear 2002). Extrapolating this result to humans suggests that the lower sensitivity to the negative consequences of alcohol that may serve as cues for adults to limit their intake, in combination with greater sensitivity to the pleasurable effects of alcohol, may encourage relatively high levels of drinking in adolescents. This effect may, in part, explain the high levels of binge drinking observed in human adolescents (Substance Abuse and Mental Health Services Administration 2003).

Of note, individuals with a family history of alcoholism also are less sensitive to alcohol, which may explain their higher risk for alcohol dependence (Schuckit et al. 2004). For children of alcoholics, this insensitivity may compound their risk for high levels of alcohol consumption during adolescence.

In addition to differences in the pharmacological effects of alcohol, adolescent animals are more vulnerable than adults to alcohol-related effects on brain plasticity and memory (White and Swartzwelder 2005), with the latter effect reported in human adolescents as well (Acheson et al. 1998). Furthermore, evidence from rodent studies using a “binge” model of alcohol exposure suggests that adolescents are more vulnerable than adults to brain damage in specific regions, including the frontal cortex (Crews et al. 2000). These studies have shown that adolescent alcohol exposure can have lasting consequences on neural and behavioral function in animals.

### Developmentally Related Effects of Alcohol Use and Exposure

#### Effects on Hormone Levels

Animal research has shown that adolescent alcohol consumption affects hormone levels. For example, acute exposure to alcohol early in adolescence increases testosterone levels in male rats (Little et al. 1992), has no effect on testosterone levels at mid-adolescence (Tentler et al. 1997), and suppresses testosterone levels in postadolescent and adult rats (Little et al. 1992; Tentler et al. 1997). Additional animal research has shown that chronic exposure to alcohol during early adolescence alters puberty-associated hormone levels and pubertal timing but that the effects are different in males and females (Cicero et al. 1990; Emanuele et al. 2002; Ferris et al. 1998; Hernandez-Gonzalez and Juarez 2000; Hiney et al. 1999; Dees et al. 1990).

#### Relationship to Future Dependence

Both prospective and retrospective human studies indicate that the early initiation of drinking is associated with later problems with alcohol, including dependence, and abuse of other substances (Grant and Dawson 1997; Labouvie et al. 1997). For example, a nationally representative survey showed that 40 percent of individuals who reported drinking before the age of 15 also described their drinking behavior at some point in their lives in a manner consistent with a diagnosis of alcohol dependence compared with 10 percent of individuals who reported starting to drink at age 21 or later (Grant and Dawson 1997). It is not clear, however, whether early alcohol use is a direct cause of later problem use or only serves as a marker for it.

Research studies using animal models have begun to explore whether a possible causal relationship exists between early alcohol exposure and later alcohol consumption and whether adolescent exposure to alcohol has lasting neuro-behavioral consequences. At this time, the findings are mixed (Spear 2002). In some studies, voluntary alcohol consumption during adolescence affected alcohol-related behavior in adult animals. For example, when the adult animals were given a choice of water or alcohol, they chose alcohol. In addition, adult animals exposed to alcohol during adolescence showed an increase in “craving” behavior, a higher probability of relapse (McBride et al. 2005), and greater alcohol intake in response to stress (Siegmund et al. 2005). In animals, chronic exposure to alcohol during adolescence induces long-lasting tolerance that “stamps in” the adolescence-associated insensitivity to the sedative (Slawecki 2002) and motor-impairing (White et al. 2000) effects of alcohol. This tolerance persists into adulthood and may contribute to high levels of adult alcohol use.

The animal research described above suggests the possibility that early exposure to alcohol may alter adolescent developmental processes, causing long-term effects that increase the propensity for later abuse. Consistent with this hypothesis, human studies show that chronic heavy alcohol use during adolescence is associated with cognitive deficits and alterations in brain activity (Tapert and Schweinsburg 2005) and morphology (De Bellis et al. 2000). It is unclear whether these neurocognitive deficits result from alcohol consumption itself or whether they were present prior to the initiation of drinking and may in fact have contributed to chronic, heavy use of alcohol (Hill 2004).

## Nonspecific Risk and Protective Factors

When considering the relationship between early alcohol use and later alcohol problems, including dependence, it is important to remember that many pathways can lead to problematic alcohol use. A broad range of specific and nonspecific factors that increase the risk of alcohol use among children and adolescents has been identified (Hawkins et al. 1992; Windle 1999). Nonspecific risk factors are those that may influence many forms of psychopathology and problem behaviors (e.g., externalizing disorders and affective and anxiety disorders) in addition to alcohol use, problem drinking, and alcohol use disorders. Specific risk factors are those that are directly related to alcohol use. No single specific or nonspecific factor can universally predict adolescent alcohol-related behaviors. Instead, combinations of factors tend to predict problematic outcomes with alcohol. Just as there are multiple risk factors for alcohol use, there are a range of protective factors that mitigate risk (Werner and Smith 1992).

### Nonspecific Risk Factors

The relative influence of nonspecific risk factors (e.g., biological, psychological, environmental, and cultural) varies both across individuals and within the same individual over time.

### Temperament, Personality, and Childhood Behavior Problems

Attributes of temperament and personality that relate to emotional reactivity and the regulation of behavior appear early, are genetically influenced, and are relatively stable. As with the birth–to–age 10 age-group, a number of these attributes, including a more difficult temperament (defined as exhibiting higher activity levels, lower task orientation, inflexibility, a withdrawal orientation, and low positive mood); high novelty seeking; high reward dependence; low harm avoidance; aggression; and behavioral undercontrol (e.g., delinquent activity, impulsivity, and difficulty inhibiting responses) predicted earlier initiation of drinking, higher levels of alcohol problems both in adolescence and in adulthood, and subsequent substance abuse and comorbid psychiatric disorders (Brown et al. 1996; Cloninger et al. 1988; Dobkin et al. 1995; Johnson et al. 1995; Tubman and Windle 1995; Zucker 2006).

#### Family Factors

Certain family characteristics are associated with higher levels of adolescent alcohol use and other problem behaviors (Hawkins et al. 1992). For example, greater marital conflict and dissatisfaction are associated with more adolescent alcohol use (Windle 1999). Similarly, stressful events and violence within the family are associated with an earlier onset of adolescent alcohol use and more frequent and heavier use (Werner and Smith 1992).

#### Dyssynchronies in Developmental Processes

Normative development refers to the concept that specific developmental processes typically occur within certain age ranges. Nonnormative development refers to development that is statistically different from that of the majority of same-age/-sex peers. Within a given age range, most variability is not likely to reflect major developmental delays or severely impact future outcomes. In some individuals, however, off-time development can contribute to nonnormative trajectories. An example that is relevant to underage drinking is the earlier sexual maturation of some girls relative to the majority of their peers. For some, cognitive and social skills may not be maturing at the same pace. As a result, these young girls, through their association with older boys, may find themselves in situations that they are not prepared to handle, such as being pressured to drink or engage in sexual activity.

### Nonspecific Protective Factors

A complex interplay of biological development, personal relationships, and physical and social environments determines an individual’s unique pathway to adulthood. As adolescents mature, they play a more active role in choosing their social relationships and physical environments, and these choices increase their risk and/or protective factors for alcohol use. An understanding of what facilitates positive pathways and outcomes and what actions, including interventions, can redirect negative pathways across the spectrum of development is critical.

#### Temperament

Just as some temperamental attributes can be risk factors for alcohol-related behaviors, others can be protective. In a longitudinal study of low socioeconomic children of alcohol-abusing parents, researchers reported that cuddly, affectionate infants and young children had a decreased risk for alcohol-related outcomes in adolescence and adulthood (Werner and Smith 1992). Children with such temperaments are thought to elicit stronger and more frequent social and emotional support, which facilitates positive development.

#### Religiosity

Religiosity often has been identified as a buffer against the early onset of, and progression to, serious alcohol involvement in adolescence. However, religiosity per se may be more of a reflection of strong family relationships and community ties rather than a protective factor itself.

#### Parenting Factors

Positive parental temperament and good parenting can buffer a child against risk for adolescent alcohol use. Four relevant domains of parenting practices have been identified, all of which may reflect the level of parental involvement and may influence the degree to which adolescents internalize parental norms, including the following (Windle et al. 2008):

- *Parental nurturance* (i.e., the level of emotional warmth and support). Higher levels of nurturance consistently are related to lower levels of adolescent alcohol use. Adolescents who view their parents as more caring, concerned, and supportive tend to delay initiation of alcohol use and consume less alcohol than adolescents who do not.
- *Parental monitoring* (i.e., establishing and enforcing reasonable rules for adolescent conduct). Higher levels of monitoring are related to lower levels of adolescent alcohol use. When parents establish explicit rules and boundaries for adolescent behavior, such as curfews and a minimum number of study hours per day, and when they reasonably and consistently enforce consequences for violating rules, adolescents tend to initiate alcohol use later and to consume alcohol less frequently.
- *Time spent together.* More time spent together by adolescents and their parents has been associated with lower levels of adolescent alcohol use.
- *Parent–adolescent communication.* Good communication has been associated with lower levels of adolescent alcohol use.

## Alcohol-Specific Risk and Protective Factors

Whereas the preceding nonspecific risk and protective factors relate to a number of problem behaviors, alcohol-specific factors are directly related to alcohol use. A variety of such factors have been identified.

### Family History of Alcoholism

A family history of alcoholism increases the risk of alcoholism in the offspring (Russell 1990). One study estimated that sons of male alcoholics are four to nine times more likely to develop an alcohol use disorder (AUD) than the sons of nonalcoholic men, whereas daughters are two to three times more likely to develop an AUD. Research studies with adoptees and twins also identified a genetic predisposition to AUDs. A family history of alcoholism also is associated with higher levels of alcohol use and deviant behavior in early adolescence and with an earlier onset of alcohol use.

Alcohol use within the family is associated with the level of adolescent alcohol use particularly when it undermines the existence of a stable, emotionally supportive family environment. In families with an alcoholic parent, inconsistency in parenting; marital conflict; spousal and child abuse; and overall stress, including financial strain, are common. These factors may contribute to earlier drinking and greater alcohol involvement by adolescents seeking to escape their home environment. Often, these youth look to a more deviant peer group to provide the social and emotional support lacking at home.

### The Influence of Siblings

Research shows that older siblings serve as role models and can influence the drinking behavior of their younger siblings. For example, a study of 508 families with an adolescent aged 11–13 and an older sibling aged 14–18 found significant associations between the older and younger siblings’ alcohol use (Needle et al. 1986). If older siblings had not used alcohol in the past year, more than 90 percent of their younger siblings reported they did not drink in the past year. If, on the other hand, the older siblings reported using alcohol 20 or more times in the past year, more than 25 percent of their younger siblings reported drinking.

### Peer Factors

Peer influences appear to result from an adolescent’s initial peer selection process and subsequent reciprocal socialization with the group. Peer selection is not a random process; rather, adolescents select a peer group based on common interests and activities. Through a series of complex interactions, they may remain with that group or move to a different one. These processes are the same whether common interests involve positive pursuits or deviant activities.

Peer influence plays a major role in adolescent alcohol use. In fact, the number or percentage of alcohol-using friends is the most potent predictor of an adolescent’s alcohol use. When a peer group experiments with alcohol or escalates its use, the peer bond of some members is strengthened, whereas other members may choose to drop out of the group.

### Development and Role of Alcohol Expectancies

Based on their experiences, people routinely form expectations about the likely consequences of their behavior, including drinking alcohol (Tolman 1932). These expectations, referred to by scientists as expectancies, influence behavior, may be positive or negative, and evolve over time (Bolles 1972; MacCorquodale and Meehl 1953; Tolman 1932).

Awareness of alcohol develops early and factors into the formation of alcohol expectancies. In one study, children as young as age 3–5 who were shown a picture of adults drinking a beverage often guessed that the adults were consuming alcohol. Those children who assumed the adults pictured were drinking alcohol were more likely 9 years later to be drinking themselves (Donovan et al. 2004). Other studies indicate that by age 9 or 10, most children have formed expectancies around using alcohol, which generally are negative ([Bibr b22-arh-32-1-30][Bibr b23-arh-32-1-30][Bibr b24-arh-32-1-30]; Kraus et al. 1994; Miller et al. 1990). Studies with slightly older children showed that they tended to endorse more positive expectancies ([Bibr b22-arh-32-1-30][Bibr b23-arh-32-1-30]; Kraus et al. 1994; Miller et al. 1990). Further, a number of studies have shown a correlation between expectancies about alcohol in early adolescence and both current and future drinking behaviors (Christiansen et al. 1989; Goldberg et al. 2002; Smith 1994; Smith et al. 1995).

Multiple factors shape adolescent expectancies, including a family history of alcoholism, parental drinking levels, early experience with alcohol, perceptions of peers’ drinking, perceived stereotypes of typical adolescent drinkers (i.e., athlete, popular student, loner, delinquent, etc.), and an individual’s prior drinking experience (Oullette et al. 1999; Smith 1994). Research with preadolescents indicates that their expectancies can be modified through focused interventions (Cruz and Dunn 2003; Kraus et al. 1994). Personality factors also influence the formation of high-risk expectancies (Anderson et al. 2003; McCarthy et al. 2001*a*,*b*; Smith and Anderson 2001; Smith et al. 2006).

## Conclusion

The period from ages 10 to 15 is characterized by dramatic changes in the adolescent’s physical, educational, and relational contexts, as well as in biological, cognitive, emotional, and social processes. During this developmental period, the child becomes an adolescent, transitions from elementary school through middle school and on to high school, and is more likely than not to have initiated alcohol use. This article reviewed some of the major developmental processes and mechanisms in this age-group as they relate to alcohol use, including peers, family, expectancies, specific and nonspecific risk and protective factors, and the effects of alcohol use on adolescent development. The following article by Brown et al. examines the period from age 16 to 20, when alcohol use peaks and the adolescent moves closer to adulthood.

## Figures and Tables

**Table:** Developmental Periods and Transitions, Key Developmental Contexts, and Developmental Tasks and Issues of Children Ages 10–15

**Variable**	**Examples**
Developmental periods and key transitions	Early adolescence –Beginnings of puberty –Transition to middle school Middle adolescence –Progression of puberty –Transition to high school
Key developmental contexts	Caregiver relationships Sibling relationships Peers and friends Intimate others Elementary, middle, and high schools Teachers, counselors, and athletic coaches Sports and physical activities Neighborhood influences and opportunities Greater contact with the larger environment including individuals outside the family and school Media (dramatic increase in involvement)
Developmental tasks and issues	Pubertal development Development of personal identity Development of ethnic and sexual identity More intimate (self-disclosing) peer relationships Beginning of dating and romantic relationships Early-onset sexual behavior, in some cases Increasing autonomy Greater self-reliance Greater self-regulation

SOURCE: Windle et al. 2008.
